# Non-Viral Therapy in COVID-19: Where Are We Standing? How Our Experience with COVID May Help Us Develop Cell Therapies for Long COVID Patients

**DOI:** 10.3390/biomedicines13081801

**Published:** 2025-07-23

**Authors:** Aitor Gonzaga, Gema Martinez-Navarrete, Loreto Macia, Marga Anton-Bonete, Gladys Cahuana, Juan R. Tejedo, Vanessa Zorrilla-Muñoz, Eduardo Fernandez-Jover, Etelvina Andreu, Cristina Eguizabal, Antonio Pérez-Martínez, Carlos Solano, Luis Manuel Hernández-Blasco, Bernat Soria

**Affiliations:** 1 Alicante Institute for Health and Biomedical Research (ISABIAL), 03010 Alicante, Spain; agonzaga@umh.es (A.G.);; 2 Institute of Bioengineering, Miguel Hernández University, 03202 Elche, Spain; 3 Nursing Department, University of Alicante, 03080 Alicante, Spain; 4 Department of Biochemistry and Molecular Biology, University Pablo de Olavide, 41013 Sevilla, Spain; 5 Biomedical Research Network for Diabetes and Related Metabolic Diseases-CIBERDEM of the Carlos III Health Institute (ISCIII), 28029 Madrid, Spain; 6 Applied Physics Department, Miguel Hernández University, 03202 Elche, Spain; 7 Advanced Therapies Unit, Basque Center for Blood Transfusion and Human Tissues, 48960 Galdakao, Spain; 8 Cell Therapy, Stem Cells and Tissues Group, Biobizkaia Health Research Institute, 48903 Barakaldo, Spain; 9 Hospital La Paz, IdiPaz, 28046 Madrid, Spain; 10 Clinic University Hospital, INCLIVA Health Research Institute, 46010 Valencia, Spain

**Keywords:** coronavirus disease 2019 (COVID-19), mesenchymal stromal cells (MSCs), cellular therapy, acute respiratory distress syndrome (ARDS), T memory lymphocytes, aging, public health, long COVID

## Abstract

**Objectives**: COVID-19, caused by the SARS-CoV-2 virus, has infected over 777 million individuals and led to approximately 7 million deaths worldwide. Despite significant efforts to develop effective therapies, treatment remains largely supportive, especially for severe complications like acute respiratory distress syndrome (ARDS). Numerous compounds from diverse pharmacological classes are currently undergoing preclinical and clinical evaluation, targeting both the virus and the host immune response. **Methods**: Despite the large number of articles published and after a preliminary attempt was published, we discarded the option of a systematic review. Instead, we have done a description of therapies with these results and a tentative mechanism of action. **Results**: Preliminary studies and early-phase clinical trials have demonstrated the potential of Mesenchymal Stem Cells (MSCs) in mitigating severe lung damage in COVID-19 patients. Previous research has shown MSCs to be effective in treating various pulmonary conditions, including acute lung injury, idiopathic pulmonary fibrosis, ARDS, asthma, chronic obstructive pulmonary disease, and lung cancer. Their ability to reduce inflammation and promote tissue repair supports their potential role in managing COVID-19-related complications. This review demonstrates the utility of MSCs in the acute phase of COVID-19 and postulates the etiopathogenic role of mitochondria in Long-COVID. Even more, their combination with other therapies is also analyzed. **Conclusions**: While the therapeutic application of MSCs in COVID-19 is still in early stages, emerging evidence suggests promising outcomes. As research advances, MSCs may become an integral part of treatment strategies for severe COVID-19, particularly in addressing immune-related lung injury and promoting recovery. However, a full pathogenic mechanism may explain or unify the complexity of signs and symptoms of Long COVID and Post-Acute Sequelae (PASC).

## 1. Introduction

Despite widespread efforts to develop efficient therapies for coronavirus disease 2019 (COVID-19) and its severe consequences, such as acute respiratory distress syndrome (ARDS), the approach to therapy has mostly remained supportive. Septic shock, hyper-inflammatory response, cytokine storm, and multiple organ failure occur in critical COVID-19 patients who develop ARDS and need intubation and mechanical ventilation [1]; however, it is not yet clearly established whether these symptoms are a direct result of viral infection or a result of the complications of critical sickness.

When COVID-19 initially arose in late 2019, treating acute infections and slowing its fast spread were the main worldwide priorities. The development of Long COVID [2,3], often referred to as post-acute sequelae of SARS-CoV-2 infection (PASC), was not anticipated, at least not in terms of scope or severity. Its emergence as an unexpected pandemic consequence has highlighted the need for long-term planning in outbreak response and the requirement for systems that can adapt to both short-term threats and long-term, chronic consequences. The treatment of any further co-infections, as well as intensive conventional supportive care, is a current clinical option.

Hypoxemia, characterized by pervasive alveolar destruction with cellular fibromyxoid exudates, significant pulmonary inflammation, pulmonary edema, and hyaline membrane development, is the primary pathologic characteristic of severe or serious COVID-19.

Mesenchymal stem (stromal) cells (MSCs), derived from different sources such as bone marrow, fat, umbilical cord, dental pulp, and placenta, have the ability to differentiate, immunomodulate, and have endogenous tissue repair capabilities. Then, MSCs display significant therapeutic possibilities for the treatment of pulmonary, cardiovascular, neurological, hepatic, and renal illnesses and are one of the most extensively studied adult stem cells in regenerative medicine. It is accepted that the immunomodulatory role of MSCs in COVID-19 is primarily dependent on regulating immune cell activation and effector function, reducing lung infiltrating cells, and promoting the clearance of pulmonary edema [4].

Furthermore, there are very few therapy choices available to individuals assisted with Extracorporeal Membrane Oxygenation (ECMO). ECMO prevents intravenous or intra-arterial MSC delivery by trapping cells in its membrane, risking circuit blockage from cell aggregation and provoking coagulation via MSC-expressed tissue factor, which together threaten ECMO function and patient safety. In a recently published paper from our group, we demonstrate that consecutive intrabronchial MSC delivery in ECMO-supported patients (CIBA-method) is both safe and feasible [5].

As the pandemic evolved, in-hospital mortality rates declined overall; however, this decline was less pronounced among aged and immunocompromised patients compared to immunocompetent individuals. The disparity was particularly evident with increasing age: immunocompromised patients with more than 80 years old were 99% for men and 98% for women, whilst for younger adults (50 to 69 years old), the risk of mortality was smaller (88% for men and 83% for women), although still quite high. Thus, despite overall improvements in clinical outcomes, immunocompromised individuals remain at disproportionately higher risk of COVID-19–related mortality. Targeted measures such as non-viral treatments, additional vaccine doses (active immunotherapy), adoptive immunotherapy (passive), monoclonal antibodies, and non-pharmaceutical ongoing reinforcement of preventive interventions are recommended for this patient population [6].

In the progression from mild to severe and critical COVID-19, there is a group of identified factors: age, obesity, type 2 diabetes, smoking, etc., that change the COVID-19 outcomes profile. Some of them are not usually well characterized, for example, Cannabis consumers. Cannabis may be a risk factor for COVID-19. Phytocannabinoids are a group of more than 100 compounds with terpenol phenolic structures (THC: tetrahydrocannabinol, CBD: Cannabidiol, CBN: Cannabinol, etc.) that interact with the endocannabinoid system in humans. Human receptors for cannabinoids affect the nervous system (CB1, receptor for psychotropic THC, and CB2 Receptor expressed in the immune system (Non-psychotropic CBD).

Based on this background, non-psychotropic cannabinoids were postulated as a tentative treatment for hyper-inflammatory reaction and cytokine storm in COVID-19. However, no clear results have been published so far. In contrast, it seems it may be useful for exhausted healthcare professionals during the COVID-19 pandemic. A randomized clinical trial in which CBD was administered twice a day (150 mg × 2) resulted in a reduction of burnout and emotional exhaustion [7]. CBD was used in the post-acute COVID symptoms (PACS) without a clear difference between the control and the treated group. Since Cannabis smokers display a mild lung inflammation, and on the other hand, cannabinoids have an anti-inflammatory function, it is worthwhile to ask which is the dominant effect of cannabinoids on COVID-19. These two sides’ effects (risk and benefit) are still an open question. Do cannabinoids increase the mortality risk of COVID-19? Or may it aid in halting the development of moderate to severe illness? [8].

For COVID-19 respiratory disease, an increasing number of clinical studies of cell-based therapies have been initiated in Spain, China, and all over the world [9,10,11]. These studies were primarily with MSCs, but they may also use conditioned media or extracellular vesicles and exosomes derived from MSCs, as well as several other cell types. Understanding the background of the research and possible MSC mechanisms of action against respiratory viral infections is crucial to designing strategies for future pandemics and long COVID.

In addition, we have reported the presence of SARS-CoV-2–specific T-cells in the memory T-cells pool (CD45RA−/CD45R0+) in the blood of convalescent donors. These CD4+ (T helper) cells support or promote the antiviral effect of CD8+ cells and can offer long-term immune protection to lessen the intensity of COVID-19 symptoms and react quickly to infection. Additionally, they may provide protection from opportunistic pathogens, provided that the donors have been in contact with during their life, which is essential to treat secondary infections that frequently arise in hospitalized patients with COVID-19 [12,13,14,15]. 

Therefore, combining MSCs and SARS-CoV-2-specific memory T (T mem) lymphocytes could offer both immediate tissue repair and durable antiviral immunity for high-risk COVID-19 patients. MSCs (or their secretome) may effectively reduce lung inflammation, stimulate regeneration through VEGF, HGF, IL-10, and TGF-β1, and also restrict fibrosis. Infused CD45RA−/CD45R0+ T memory cells activate virus-specific cytotoxic and helper functions, accelerating viral clearance and providing long-term protection against SARS-CoV-2 and secondary pathogens. Together, MSCs mitigate the dysregulated inflammation that drives mortality in immunocompromised patients, while CD45RA-/CD45R0+ T memory lymphocytes restore adaptive antiviral defenses, an approach that is especially useful for the elderly or immunosuppressed populations who remain at risk despite vaccination and standard therapies.

## 2. Long COVID

**Figure 1:**
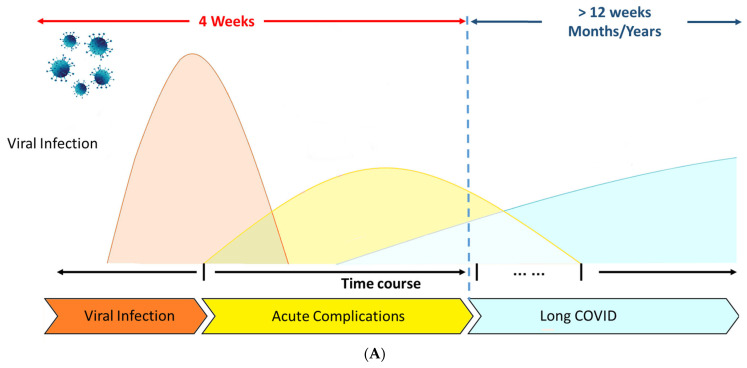

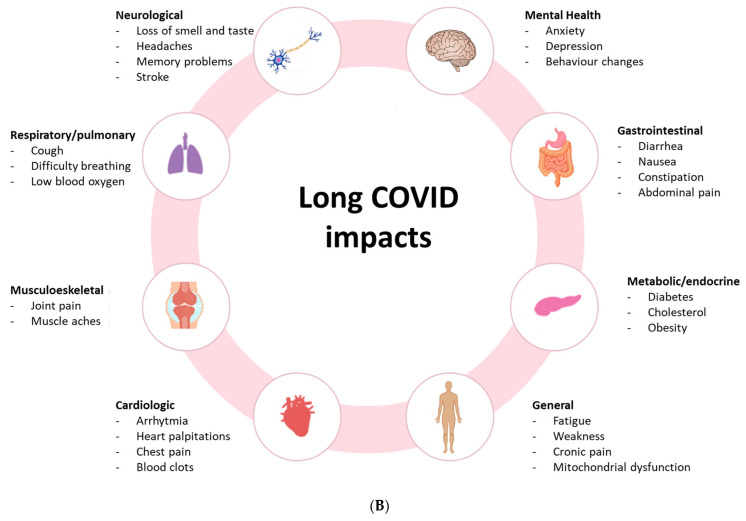
(**A**) COVID-19 and Long COVID. Modified from Li J., et al. [29]. (**B**) Common symptoms associated with Long COVID and possible underlying pathophysiology. Modified from Liew F., et al. [30].

Long COVID is characterized by COVID-19 symptoms that “last for more than 12 weeks following the onset of acute symptoms and cannot be linked to any other illnesses”. The World Health Organization (WHO) defines the Long COVID condition as “the illness that occurs in people with a history of probable or confirmed SARS-CoV-2 infection; typically within 3 months of the onset of COVID-19, with symptoms and effects that last for at least 2 months”(World Health Organization. Coronavirus disease (COVID-19): post COVID-19 condition). Figure 1A describes the time course that follows this complex symptomatology.

A recent review of Long COVID symptoms in children and teenagers found that the frequency of fatigue ranged from 3% to 87%, while a meta-analysis found that the range of the prevalence of fatigue was 32% to 62% [16,17]. However, the variety of symptoms (Figure 1B) impacts almost every system, from neurological to respiratory, gastrointestinal, metabolic, musculoskeletal, cardiologic, and general. A subgroup of cases that experience sequelae following a moderate acute infection may be classified as having Post-Infective Fatigue Syndrome (PIFS), which is characterized by enduring symptoms and disability along with few results on a routine clinical evaluation [18,19,20,21]. Both PIFS and PCC’s fundamental disease processes are still unknown. The spectrum of hypothesized causes for PIFS includes low-grade inflammation to functional changes in the brain’s sense of bodily conditions that are partially brought on by psychosocial variables [22,23]. A recent meta-analysis found 41% increased odds of Long COVID with pre-existing asthma and 32% with pre-existing Chronic Obstructive Pulmonary Disease (COPD) [24].

Although the precise mechanisms behind Long COVID remain unclear, there is growing evidence that autoimmunity may be involved in certain cases. The immune system, which was initially activated to fight the SARS-CoV-2 virus during the acute sickness, is thought to have the potential to become dysregulated and turn against the body’s own tissues and cells [25,26,27,28].

**Figure 2:**
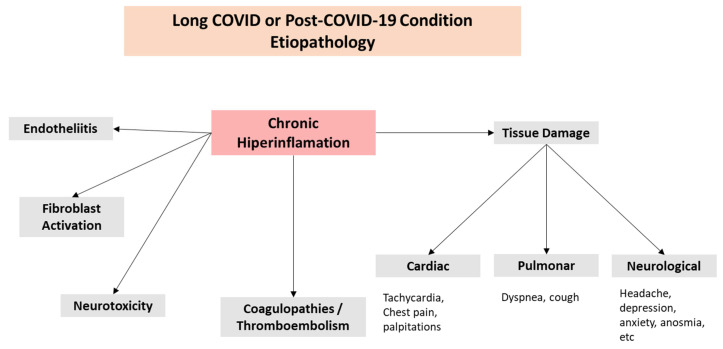
Long-COVID complications.

Although several variables have been suggested (Figure 1B), the possible processes that may contribute to the pathophysiology of Long COVID are still unknown (Figure 1A). The existence of a persistent hyperinflammatory condition could be a crucial factor [31]. Given that the most often impacted organ by SARS-CoV-2 infection is the lung, severe COVID-19 is frequently followed by chronic respiratory symptoms and fitness restrictions. Dyspnea is the most common chronic respiratory condition [32], followed by cough and chest discomfort [33,34]. The virus triggers natural immunity at the lung level, which causes the production of inflammatory cytokines like interleukin (IL)-6 (IL-6), IL-1, TNF-α, and reactive oxygen species (ROS). As a result of endothelial injury brought on by the stimulation of fibroblasts with deposits of collagen and fibronectin, these systemic increases of cytokines have been linked to the development of pulmonary fibrosis as well as heart and neurological disorders (Figure 2).

Following the initial illness, some people experience chronic symptoms for weeks to months. The underlying etiopathology of Long COVID comprises several variables (Table 1). Among the current therapies, metformin has the greatest clinical evidence, as indicated by two major phase 3 studies that showed significant decreases in the incidence of Long COVID (42% to 63%) [35,36]. This will support the role of mitochondria, since metformin acts by inhibiting mitochondrial complex I and adenosine monophosphate-activated protein kinase (AMPK). Emerging therapies for Long COVID address chronic inflammation, immunological dysregulation, and hormonal abnormalities, among others linked with the illness (Table 1).

### 2.1. COVID-19

Aging-like symptoms. Long-COVID and aging share etiopathogenic mechanisms such as persistent inflammatory response with elevated IL-6 and TNFα, immunosenescence with exhausted lymphocytes, mitochondrial dysfunction with increased reactive oxygen species (ROS) [48], stem-cell exhaustion impairing lung, cardiac, and neural repair, endothelial senescence promoting microthrombi, and altered epigenetic patterns resembling advanced biological age, creating a vicious cycle in which SARS-CoV-2 both exploits and accelerates age-related decline [49]. Note that MSC treatment seems to be more effective in older patients, a hypothesis that may need further research.

According to previous research from Leng et al. [50], a single intravenous infusion of ACE2-mesenchymal stem cells produced a remarkable recovery even in a critically ill elderly COVID-19 patient. The patient was discharged within 10 days, pulmonary function and symptoms improved within 2 days, and overactivated cytokine-secreting immune cells disappeared within 3–6 days. This is truly remarkable considering the patient’s age and initial severity. In favor of this interpretation is the fact that cell therapies with MSC or CD45RA- increase the lymphoid to myeloid ratio coming back to a younger profile [11,14].

### 2.2. Epigenetic Changes

COVID-19 infection also causes substantial acute and long-term implications, including changes in cellular memory. It has been demonstrated that the methylation of certain CpG sites may identify COVID-19-infected individuals from healthy controls [51,52,53]. A large number of research articles have looked at the acute and long-term effects of COVID-19 on health. Measuring DNA methylation-based aging in healthy and COVID-19-infected participants indicated that COVID-19 infection accelerates epigenetic aging and telomere attrition. Additionally, accelerated epigenetic aging is linked to an increased likelihood of SARS-CoV-2 infection and the development of severe COVID-19 [54,55].

Furthermore, COVID-19 infection promotes DNA methylation-based aging rates. This finding implies that environmental variables such as COVID-19 infections have long-term impacts on DNA methylation, which may influence the adaptive immune response to future illnesses or the expression of major genes related to aging. It is feasible that epigenetic clocks can monitor post-COVID recovery, at least in terms of age acceleration [56].

### 2.3. Immune Response Changes

Aging causes a change in the primary stimulatory mechanisms that mediate the B-cell response to antigens, a shift from naïve to memory B cells, and a decrease in the ability of antigen recognition sites on antibodies to identify new infections. Furthermore, long-lived plasma cells decline in response to antiviral vaccinations [57]. Furthermore, in addition to a reduced number of naïve T-cells, there is also diminished interaction between T-cells and antigen-presenting cells, a critical process required for the differentiation of naïve T-cells into memory cells [58].

## 3. Antiviral Therapies

**Figure 3:**
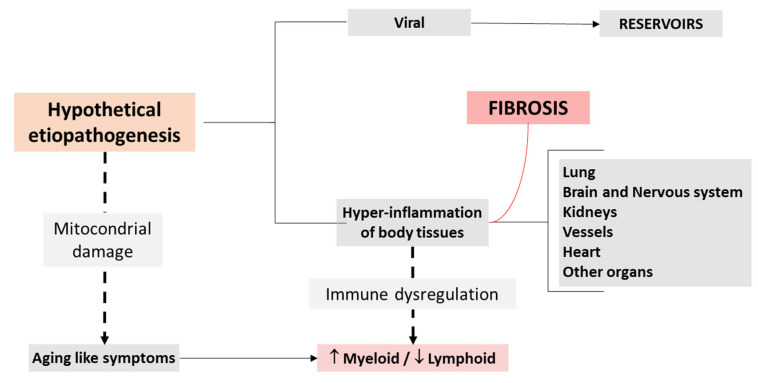
Etiopathogenic simplified model.

SARS-CoV-2 infection has been linked to over 100 different symptoms [59], reflecting its ability to affect virtually every organ system in the body—including the cardiorespiratory, gastrointestinal, neurological, dermatological, musculoskeletal, endocrine, visual, and reproductive systems. Notably, research indicates that individuals with long COVID often report symptoms predominantly related to a specific system. This pattern has led to the hypothesis that distinct symptom clusters or subgroups exist within long COVID. These clusters may represent different underlying mechanisms and pathophysiological processes (Figure 3).

The therapy landscape for COVID-19 has shifted dramatically, with antiviral medications remaining the cornerstone for early-stage infection in high-risk individuals. During the initial phase of infection—characterized by high viral load and a limited adaptive immune response—interventions that inhibit viral replication are most effective. These include direct-acting antivirals such as nirmatrelvir/ritonavir, molnupiravir, and remdesivir, as well as passive immunotherapies, including anti-SARS-CoV-2 monoclonal antibodies, convalescent plasma from recovered donors, and adoptive immunotherapy strategies.

Nirmatrelvir-ritonavir, a combination of oral protease inhibitors, is a COVID-19-specific therapeutic intervention for symptomatic outpatients at risk of progression to severe disease. It has been shown to significantly reduce the likelihood of hospitalization and mortality in individuals with mild to moderate COVID-19 (i.e., no hypoxia). Nirmatrelvir-ritonavir should be initiated as soon as possible following COVID-19 diagnosis and within five days of symptom onset, lowering hospitalization and mortality rates by 87% when started within 5 days of symptom onset; nevertheless, medication interactions and rebound cases must be carefully managed [60].

Although the antiviral agent molnupiravir has an EUA (Emergency Use Authorization) in the USA, for mild to moderate symptomatic COVID-19 in patients at risk for progression and is recommended by some guidelines (e.g., National Institute of Health), its benefit is unproven, and studies do not consistently demonstrate efficacy against hospitalization or death.

Remdesivir has been shown to reduce COVID-19–related hospitalizations; however, its administration—requiring three intravenous (IV) infusions over three consecutive days—presents logistical challenges in outpatient settings. As such, remdesivir may be more appropriate for patients residing in institutional environments, such as skilled nursing facilities. High-titer convalescent plasma represents another potential therapeutic option.

Based on findings from clinical research carried out in the early phases of the COVID-19 pandemic, high-titer use of convalescent plasma proved effective when administered early after symptom start (<72 h), when the viral load is relatively low, and before inflammatory damage is noticeable [61], although treatment did not shorten hospital stays, it increased the probability of clinical recovery [62,63]. While it has demonstrated efficacy in reducing COVID-19–associated hospitalization, its use is limited by the need for donor screening, plasma collection, and antibody quantification processes, which may constrain widespread availability. Even more, clinical outcomes do not support this option. The use of low-titer convalescent plasma is not appropriate or authorized by the FDA using the Emergency Use Authorization (EUA) [63].

At this stage, Non-Antiviral therapies have been shown to be moderately useful. For patients who require mechanical ventilation or ECMO, so far, the recommended treatment is low-dose dexamethasone; for those who are within 24 to 48 h of admission to an ICU and within 96 h of hospitalization, the addition of tocilizumab or baricitinib may improve mortality. Even more, a recent report from our group has solved the method to apply cell therapies in ECMO Patients using Wharton jelly MSC (CIBA method) [5]. Cell therapy represents a useful tool in decreasing the hyper-inflammatory response and preventing Long COVID complications, a conclusion that will need retrospective studies.

Antiviral treatments must be started as soon as possible since they work best when administered five to seven days after the onset of symptoms. An excessive and abnormal inflammatory response is thought to be the main source of immunopathological damage later in the course of the disease, particularly in patients with severe and particularly critical conditions. Anti-inflammatory treatments such as low-dose corticosteroids, IL-6 inhibitors, or JAK inhibitors have shown advantages at this point (COVID-19 Guidelines, last accessed 7 February 2023).

There are no monoclonal antibodies that are active against the increasingly prevalent Omicron sublineages BQ.1 and BQ1.1 in the US, and the efficacy of monoclonal antibodies varies against the many SARS-CoV-2 variants [64]. Therefore, bebtelovimab is no longer approved for use in the US to treat COVID-19 [65,66]; however, in situations when SARS-CoV-2 variations that are sensitive to monoclonal antibodies are highly prevalent, monoclonal antibodies can still be regarded as a sensible substitute.

While antiviral therapies might slow the course of Post-Acute Sequelae SARS-CoV-2 infection symptoms if given early on, non-antiviral treatments may provide broader symptom relief by treating immunological dysregulation, inflammation, and other complicated pathophysiological components of long COVID. For the overall management of long COVID, non-antiviral therapies—especially those that target immunological and inflammatory responses—prove to be beneficial, perhaps boosting or supplementing the benefits of antiviral therapy, thus offering a multifaceted approach that goes beyond early viral suppression.

These antiviral treatments are critical components of the current COVID-19 therapeutic strategy. Their effectiveness varies depending on vaccination status, administration time, and individual patient characteristics. Healthcare practitioners use these characteristics to select the best therapeutic approach for each patient [67].

In addition to antiviral therapies, immunomodulatory treatments such as corticosteroids (e.g., dexamethasone), interleukin-6 receptor antagonists (e.g., tocilizumab), and Janus kinase inhibitors (e.g., baricitinib) have been effective in reducing mortality by mitigating the hyperinflammatory response seen in severe COVID-19 cases [68].

## 4. Immunotherapy with CD45RA−/CD45R0+: CD45RA Depleted/CD45R0+ T-Cells: More Than Antiviral

It is still unclear how adaptive immunity functions in COVID-19, how T-cells provide protective immunity, and how memory T-cells contribute to COVID-19 protection [13,69,70].

The function of adaptive immunity in COVID-19, particularly T-cells and memory T-cells, is being studied extensively, and it offers great promise for understanding long-term protection against COVID-19. T-cells play a key role in the immune response, with CD4⁺ helper T-cells promoting adaptive immunity [71], B-lymphocytes producing antibodies, and CD8⁺ cytotoxic T-cells destroying virus-infected cells. Robust T-cell responses have been found in individuals with moderate or silent infections, showing that even in the absence of large antibody titers, T-cell-mediated immunity may contribute to successful viral clearance and long-term protection [72].

Furthermore, memory T-cells, which survive for months after the original infection or vaccination, are essential for fast reactivation upon re-exposure to the virus. These cells have been demonstrated to identify conserved viral epitopes, providing cross-protection against new variations even when antibody responses decline [73].

Memory T-cells arise following the detection of a pathogen by local antigen-presenting cells. This triggers the activation and expansion of T-cells, which then become effector cells that release cytokines to help eliminate the infection. Once the threat is resolved, most of these responsive T-cells die off, leaving behind a diverse, long-lasting population of memory T-cells poised for future immune challenges [74,75]. This memory T-cells population, designated as CD45RA− or CD45R0+, is maintained throughout time, providing immediate and long-term immunological protection against recurrent reinfections [76,77].

In a previous work from our group, we describe and develop techniques for isolating, banking, and growing SARS-CoV-2-specific memory T lymphocytes (T mem) from convalescent COVID-19 donors as a possible “off-the-shelf” adoptive cell treatment [13]. In regard to memory T-cells, infusing them would enhance their number in chosen individuals who have low levels owing to viral infection. These cells have long-life memory and, upon re-encountering SARS-CoV-2, would elicit an improved effector function, leading to better patient protection [78,79].

## 5. Combinatorial Cell—Therapies: MSC and CD45RA Depleted Memory T-Cells Trial

T-cell exhaustion was initially characterized by the clonal deletion of CD8 T-cells specific to viruses during prolonged and intense chronic infections [80]. It is now evident that T-cells may not undergo physical deletion in the presence of persistent antigens; instead, they can lose functionality and become incapable of executing the typical range of effector activities associated with robust and protective effector and memory T-cell populations [81]. Exhaustion is not confined to CD8 T-cell responses; CD4 T-cells have also demonstrated functional unresponsiveness in the aftermath of various infections [82,83,84,85].

T-cell exhaustion is characterized by the progressive loss of effector functions and the upregulation of several inhibitory receptors (IRs), such as programmed cell death protein 1 (PD-1), T-cell immunoglobulin domain and mucin domain-3 (TIM-3), lymphocyte activation gene-3 (LAG-3), and T-cell immune receptor with Ig and ITIM domains (TIGIT). Reduced proliferation and differentiation, weaker cytokine responses, particularly those of interleukin (IL)-2, tumor necrosis factor-α, and interferon-γ, a changed cellular metabolic profile, a modified cellular gene—expression profile, and a shifting epigenetic profile are other indicators of this fatigue [86,87,88,89].

The large number of trials conducted until now demonstrates that cell therapy has been recommended as a helpful approach to managing COVID-19 and its complications, such as ARDS. With the main goal of reducing morbidity and death rates by enhancing host immunity, reducing inflammation, and minimizing lung damage, novel treatments for COVID-19 are currently being investigated. According to earlier clinical studies, cell treatment is safe and has a variety of positive benefits on a variety of respiratory disorders [90,91,92].

Pro-inflammatory mediators were significantly reduced after the infusion of adipose tissue-derived MSC (a-MSC) and UC-MSCs, according to two pioneering studies. In the former, the control group consisted of 29 patients, whereas the UC-MSC therapy was administered to a total of 12 individuals. Both groups received antivirals and glucocorticoids prior to the experiment. After day 3 of stem cell infusion, the C-reactive protein (CRP) and IL-6 levels in the a-MSC and UC-MSC groups were considerably lower than those in the control group. Lymphopenia was recovered with higher CD4 and CD8+ counts (a-MSC). In both groups, time to clinical improvement was quicker, and chest CT scans revealed that patients in that group had less lung inflammation than those in the control group. Improvement was evident following MSC administration, even if variations in mortality between the UC-MSC group did not achieve statistical significance [93].

Earlier studies revealed that SARS-CoV-2-specific CD45RA-memory T-cells from the blood of recovering donors include a population that is simple, effective, and easy to separate. Therefore, they may be able to remove virally infected cells and bestow T-cell immunity [13], most probably reactivating the innate-adaptive equilibrium in the immune response.

Advanced Therapies Medicinal Products (ATMPs) are living medicaments, must be produced in certified Good Manufacturing Practice facilities, and used by healthcare specialists trained in the use of cellular medicaments. The quality of the product, free of side effects [94,95,96] and proper administration protocols, results in better clinical outcomes. We anticipate that cell-based therapy with anti-inflammatory and pro-regenerative MSCs may ablate COVID-19 lung and systemic complications and long-term sequelae.

### 5.1. Mesenchymal Stromal Cells

Clinical investigations and basic medical research have placed a strong emphasis on MSC, which is thought to be the “physiological” medication that heals the body in adults. Initially, it was thought that MSCs would go to the sites of injury, engraft, and differentiate into functional cells, resulting in the repair of damaged or diseased connective tissues. In initial investigations, it was suggested that MSCs possess the capability to migrate towards areas of injury, subsequently undergoing differentiation into functional cells committed to restoring tissue functionality [97]. Nevertheless, findings from several hundred human trials and animal research undertaken over the past few decades have questioned this traditional paradigm. In summary, despite the fact that MSCs were proven to be remarkably effective in a number of disease models, it became clear that the cells did not engraft in enough individuals or for long enough to fully account for the outcomes in terms of tissue restoration [98,99,100]. More unexpectedly, MSCs could even engraft and differentiate into functional cells of tissues that did not develop from mesoderm [101,102], challenging the long—held belief that adult stem cells can only normally differentiate into tissues that come from their origin [103,104].

The conundrum of effectiveness without long—term engraftment, particularly in non-mesodermal tissues, is still largely unresolved and a subject of intense discussion [105,106] and cannot be considered as a mechanism of action for COVID-19 beneficial effects. In contrast, new observations suggest that MSCs repair damaged and ill tissues/organs using alternative modes of functional rescue and repair that increase cell viability and/or proliferation, decrease cell apoptosis, and, in some cases, modulate immune responses. This is in conflict with the long—term engraftment and differentiation that was previously believed to be the mechanism by which MSCs heal diseased and injured tissues/organs.

MSCs may operate through a variety of mechanisms (Table 2), several potential mechanisms through which they confer their advantageous effects have been postulated, each with significant implications for therapeutic applications; (a) Differentiation and Tissue Integration: MSCs can differentiate into specific cell types, such as osteoblasts, chondrocytes, or adipocytes, allowing them to integrate seamlessly into damaged tissues. This property holds great promise for regenerative medicine, where replacing or repairing damaged tissues is a primary objective. (b) Efferocytosis: Currently, in-vivo differentiation does not seem to be the therapeutic mechanism; instead, macrophages engulf MSCs in a process called efferocytosis [107]; (c) another proposed mechanism involved the fusion of MSCs with impaired cells, facilitating the regeneration of damaged tissues [108,109]; (d) Secretion of Bioactive Molecules: MSCs actively secrete a diverse range of bioactive substances, including cytokines, growth factors, and extracellular vesicles like exosomes. These molecules play crucial roles in supporting cell proliferation, promoting tissue repair, enhancing cell survival, and modulating inflammatory responses. This paracrine activity contributes significantly to the therapeutic efficacy of MSCs, particularly in inflammatory and degenerative conditions, and (e) Direct Cellular Interaction: Through direct physical contact with host cells, MSCs can influence the behavior and function of effector cells, such as immune cells. This interaction can lead to the modulation of immune responses, suppression of inflammation, and enhancement of tissue repair processes [110].

These mechanisms underline the multifaceted therapeutic potential of MSCs, making them a cornerstone of advanced research in tissue engineering, immunomodulation, and regenerative medicine.

### 5.2. Secretome

The secretome of MSCs exhibits dynamic characteristics that vary according to culture conditions, which have specific therapeutic implications for COVID-19 treatment. The general features of the secretome include a multimodal composition comprising soluble factors such as VEGF, FGF, IL-10, and TGF-β, along with extracellular vesicles consisting of exosomes enriched in microRNAs and tetraspanins (CD63/CD81). The paracrine action mechanism of the secretome regulates inflammation, promotes tissue regeneration, and modulates immune responses through interactions with Toll—like receptors (TLR3/TLR9).

Tentative use in COVID-19 is supported by the fact that the secretome has been observed to suppress cytokine storms by reducing IL-6, TNF-α, and MCP-1 levels by 40–60% while elevating IL-10 concentrations. Additionally, it facilitates the polarization of macrophages from M1 to M2 phenotypes and increases regulatory T-cells. Direct protection is conferred by the presence of β-defensins, LL-37 cathelicidin, and hepcidin, which inhibit viral entry, alongside exosomes rich in microRNAs (miR-21, miR-146a) that block SARS-CoV-2 replication. Moreover, the secretome will aid pulmonary repair by restoring the alveolar-capillary barrier via angiopoietin—1 and reducing fibrosis through the matrix metalloproteinases (MMPs) regulation and their inhibitors (TIMPs).

Several studies have explored the potential of MSCs’ secretome as a therapeutic approach for COVID-19, emphasizing its immunomodulatory and regenerative benefits [111,112,113,114]. Current clinical trials, such as NCT04753476 and NCT05122234, which examine the safety and effectiveness of MSC secretome—based therapy in COVID-19 patients, provide additional support for these findings. All of these initiatives point to the possibility that the MSC secretome might provide a new, cell-free treatment approach for COVID-19, especially in more severe instances.

Recent advancements in research have unveiled additional facets of MSC functionality. Studies now underscore the significance of paracrine factors [99,115]—substances secreted by MSCs influencing nearby cells—implying a crucial role in mediating their therapeutic effects. Moreover, the exchange of mitochondria, known as mitochondrial transfer [116], has emerged as a noteworthy mechanism by which MSCs contribute to tissue repair and regeneration. Furthermore, the secretion of extracellular vesicles [117] containing bioactive molecules, microRNAs, and proteins has been identified as a pivotal mode through which MSCs communicate with neighboring cells and exert their beneficial impact. This evolving understanding of MSC biology emphasizes the intricate interplay of various mechanisms, from cell migration and differentiation to paracrine signaling, mitochondrial transfer, and extracellular vesicle secretion, collectively contributing to the overall therapeutic potential of MSCs.

One important characteristic of MSCs has been shown to be their potential to regulate immunological responses. Several research projects have demonstrated that MSCs have potent immunosuppressive effects by blocking the activity of both innate and adaptive immune cells [118,119]. MSCs specifically release cytokines including IL-6 and IL-13, prostaglandin E2, keratinocyte growth factor, and granulocyte—macrophage colony—stimulating factor, which can affect how innate and adaptive immune cells interact with the cellular environment. These soluble components can influence the cytokine profile produced by immune cells and encourage alveolar macrophage phagocytosis [107]. These processes may be efficient enough for respiratory infections [120]. Furthermore, preclinical research strongly suggests that MSCs and their secretomes might be thought of as novel and efficient treatment against ARDS [121,122].

## 6. MSC Mechanism

The use of MSC derived from adipose tissue has been linked to clinical improvement in COVID-19 patients who are severely sick (Grade 6 to 7, intubated and mechanically ventilated ICU patients), as shown in earlier research in a pilot study by our lab [11] which has been confirmed by numerous Phase 2 clinical trials, as highlighted in a recently published systematic review, a meta-analysis of 24 studies employing intravenous (IV) MSCs revealed that MSC therapy was associated with a decreased risk of all—cause mortality [10]. The Relative Risk (RR) was calculated as 0.63 [95% CI 0.46 to 0.85] (*p* < 0.01), and the Odds Ratio (OR) stood at 0.51 [95% CI 0.33 to 0.78] (*p* < 0.01). This underscores the potential efficacy of MSC therapy in mitigating mortality across the analyzed studies [10].

We could also demonstrate that MSC infusion had no side effects in these critically ill patients with respiratory failure, severe inflammation, and strong prothrombotic risk. The unexpected COVID-19 emergency stimulated numerous trials [123], most of the time with insufficient preclinical basis [124]; however despite the initial skepticism, MSC and memory T-cells remain as providers of cell therapy for vulnerable critical patients whilst others (hydroxyquinine, ivermectin) are discarded on the basis of clinical results or with limited antiviral efficacy. This review will explore non-antiviral cell therapy options to treat COVID-19 patients.

### 6.1. MSC Mechanism of Action (MoA)

The interest in the use of MSCs in cell therapy is mainly based on their mechanism of action. The desired therapeutic effect depends on many factors since the mechanism of action of ATMPs is likely to be multifaceted.

MSCs can differentiate into a variety of mesenchymal tissues, such as bones, cartilage, fat, muscles, tendons, and bone marrow, both in vivo and in vitro. Additionally, MSCs have the ability to transdifferentiate, producing cells with traits from several lineages, such as neuron-like cells, hepatocytes, epithelial-like cells, and pancreatic islet-like cells (non-mesodermal cells). Furthermore, MSCs secrete a diverse range of pro-inflammatory and anti-inflammatory cytokines, chemokines, growth factors, and prostaglandins both under resting and inflammatory conditions [125]. These molecules are associated with:

1. Immunomodulation: transforming growth factor beta (TGF-β), human leukocyte antigen-G5 (HLA-G5), hepatocyte growth factor (HGF), indoleamine-2,3-dioxygenase (IDO), and prostaglandin E2 (PGE-2).
2. Anti-apoptotic factors include vascular endothelial growth factor (VEGF), insulin-like growth factor 1 (IGF-1), granulocyte-macrophage colony-stimulating factor (GM-CSF), TGF-β, and stanniocalcin-1 (STC1).
3. Angiogenesis: monocyte chemoattractant protein 1 (MCP-1), IGF-1, and Vascular Endothelial Growth Factor (VEGF).
4. Angiopoietin-1, stromal cell-derived factor 1 (SDF-1), and SCF complex enhance the proliferation and development of local stem and progenitor cells.
5. HGF and basic fibroblast growth factor (bFGF), which prevent fibrosis and
6. C-C motif chemokine ligands 2 and 4 (CCL2, CCL4) and C-X-C motif chemokine 12 (CXCL12, also known as SDF1) are involved in chemoattraction [126].

MSCs are constitutively negative for HLA class II and show low expression of the major histocompatibility complex class I human leukocyte antigen (MHC-HLA class I). Furthermore, co-stimulatory molecules, including CD80, CD86, CD40, and CD40L, are not expressed by them. However, MSCs do express surface markers, including vascular cell adhesion protein 1 (VCAM-1), intercellular adhesion molecule 2 (ICAM-2), and lymphocyte function-associated antigen 3 (LFA-3 or CD58), which are also found on thymic epithelial cells and play a critical role in T cell interaction [127]. The immunomodulatory properties of MSC are not strictly related to immunosuppression. While MSCs are in a quiescent state, exhibiting antiapoptotic qualities and aiding in homeostasis, they start to use their immunomodulatory skills in an inflammatory environment (IFNγ, TNFα, IL-1α, and IL-1β) to prevent the growth of effector cells and the production of their cytokines. In the same way, MSCs can block a variety of immune cell functions. It has also been proposed that MSCs interact with the environment by releasing pro-inflammatory compounds when the level of inflammatory cytokines is low [128].

### 6.2. Homing Mechanism

Previous studies suggest that the therapeutic abilities of MSCs play a functional role at sites of tissue damage and inflammation [129]. By modifying the local immune cells to prevent them from monitoring the injured tissue, these therapeutic actions prevent the onset of autoimmune reactions [130]. The mechanism by which MSCs arrive at the injured tissue is not fully understood, but it has been demonstrated that MSCs migrate to damaged tissue exhibiting inflammation [131]. MSCs adhere to vascular endothelial cells and undergo a trans-endothelial migration, which consists of crossing the endothelial barrier [132]. The migration process is highly dependent on the chemokine receptor CXCR4 and its binding partner, SDF-1, CXCL12 [133]. However, most of the cells are trapped in the microvasculature of the lung [134].

The ability of injected cells to migrate, survive, integrate, and create useful paracrine mediator factors (also known as “cell-cell interaction”) can be used to assess the efficacy of cell therapy. Advanced Therapy Medicinal Products’ (ATMPs) phenotype and therapeutic qualities are impacted by several disorders. The recipient tissue must react favorably to administering ATMP in order for endogenous regeneration processes to be activated, which is necessary in the pursuit of safety and effectiveness [135]. Understanding the integration of the exogenous mechanisms (injected ATMP) with the endogenous recipient (host) will play a decisive role in the future clinical use of adult stem cells [136].

## 7. Clinical Trials with MSC

Advanced Therapy Medicinal Products (ATMPs) represent a cutting-edge class of biomedical treatments encompassing gene therapy, somatic cell therapy, and tissue engineering. Among them, MSCs, a subset of adult stem cells, have shown great promise because of their capacity for various cell lineage differentiation, immunomodulatory effects, and regenerative capabilities.

In July 2025, a thorough search was carried out using the query phrases “Mesenchymal Stem Cells” and “Mesenchymal Stromal Cells” in the publicly accessible clinicaltrial.gov database in order to evaluate the clinical landscape and safety profile of MSC-based therapeutics. A total of 465 registered clinical studies examining the use of MSCs in diverse therapeutic situations were found through this search. The constant positive safety profile of MSCs observed by these investigations supports their ongoing research and application in ATMP-based treatments.

In order to evaluate the current state of clinical studies examining the application of MSCs in the treatment of COVID-19, another comprehensive search was carried out. In order to search the publicly accessible clinical trial database (clinicaltrials.gov), the combined query phrases “Mesenchymal Stem Cells” AND “Mesenchymal Stromal Cells” AND “COVID-19” were used.

A total of 36 clinical studies assessing MSC-based treatments for COVID-19 were found through the search. Of these, 11 trials were finished, 2 were still in progress but not recruiting, 6 were marked as withdrawn or halted, 3 were ended, 1 trial was still in progress, and 13 were classified as having uncertain status. At the time of study, none of these studies had advanced to Phase 4, and the bulk were still in early clinical phases (Phase 1 and Phase 2).

Since MSCs are able to in-vitro limit proliferation and alter the actions of both innate and adaptive immune cells, it is most likely that MSC treatment can block the immune system’s storm release of cytokines and boost endogenous repair [137], however, more studies are needed to explore the behavior of these “therapeutic cells” when face an injured—infected tissue. Our previous work showed that an MSC did not block the proliferation of T-lymphocytes in critical COVID-19 patients. In contrast, T-cell counts recovered after an MSC administration [11].

Through the use of a paracrine mechanism of action [138], MSC therapies operate by modulating inflammatory disease responses. They relocate to inflammatory and damaged sites so they can exert their immunomodulatory actions [139]. Its immunomodulatory action stems from its capacity to suppress B, T, and NK cell growth and activity in addition to dendritic cell activity, as well as the differentiation of monocytes into anti-inflammatory macrophages, effector T-cells into regulatory T-cells, and cytokine secretion [140].

The unexpected onset of the COVID-19 pandemic strained the ability of the health systems in many of the impacted nations since many of these patients required extensive care [141]. Mortality rates for patients hospitalized in the intensive care unit (ICU) who need mechanical ventilation are quite high, ranging from 30 to 60% [142]. Even in those patients with a favorable outcome, an additional problem that contributes to the ICU saturation is the long average stay under invasive mechanical ventilation [143]. Therefore, any adjuvant treatment that contributes to accelerating patient recovery will represent a major step forward. At this point, as stated by Khoury et al. [124], information published on critically ill patients undergoing mechanical ventilation treated with MSCs was reduced to a single patient under mechanical ventilation, also reported by Leng Z et al. [50], then, Prof. Soria, from the University Miguel Hernández and ISABIAL-Alicante and Garcia-Olmo from Autonomous University of Madrid and Fundación Jimenez Díaz convinced the rest of the members of the Spanish Cell Therapy Network (Red TerCel) to use our expertise [144] and MSC doses already in our biobanks to detain this unbearable attack on the healthcare system. Important to note that at this moment, the International Society for Cell Therapy experts recommended “not to begin MSC clinical trials on COVID-19” [145].

The early-pandemic mindset, when cautious suggestions were inspired by doubts over the safety and effectiveness of new therapies. In particular, it pointed out that although MSCs had demonstrated potential in instances of ARDS in general, their application in ARDS caused by viruses, such as COVID-19, had not yet been validated. In order to gather further information, some authors suggested halting these experiments [145]; however, despite this caution, the urgency of the COVID-19 pandemic catalyzed a surge in MSC clinical trials.

In this context, the BALMYS-19 Clinical Trial (BAttLe against COVID-19 using Mesenchymal Stromal cells; EudraCT: 2020-001266-11; NCT04348461, PIs: Bernat Soria and Damian García-Olmo) was approved by the Spanish Agency for Medicaments (AEMPS) and was initiated. Needless to say, the basis was more theoretical than practical; in fact, only one critically ill patient was described in the literature [50]. In the pilot study [11], 13 WHO Grade 6–7 patients (critically ill and mechanically ventilated COVID19 patients) were treated with 1 × 10^6^ cells/kg of weight of adipose tissue-derived MSCs (a-MSC) in one or several doses as compassionate use, as they did not respond to conventional treatment (prior use of steroids, lopinavir/ritonavir, hydroxychloroquine, and/or tocilizumab, among other antiviral and/or anti-inflammatory medications). Patients from saturated ICUS that did not respond to the WHO-recommended treatments (included in the list of the SOLIDARITY studies) were treated from 3 April to 23 April 2020. In summary, the treatment was safe and associated with clinical, radiological, and biological improvements (e.g., reduction in serum levels of CRP, IL-6, ferritin, LDH, and D-dimer as well as an increase in B- and T-lymphocyte counts). The first dose of MSCs was administered at 7 days average (range 1–30) after mechanical ventilation, patients receiving the cellular medicament earlier have a better outcome, an statement established by a single WHO Grade 4 case patient treated with our technique (Confidentiality Agreement with Prof B. Soria, dated 29 March 2020) [126]. Despite the limited number of cases, this was the largest cohort of critically ill patients published at that time within the BALMYS Clinical Trial (intubated with mechanical ventilation). Mortality decreased from 70–85% (previously reported for this group of patients) to 15% (Exitus: “2 patients” was not related to MSCs infusion). These positive but preliminary results could be attributed to: a direct effect of MSC on the COVID-19 lung complications pathophysiological mechanisms; the quality and safety of the medicinal product, absence of prothrombotic effects, and the administration route and protocol. The downregulation of pro-inflammatory cytokine expression and a rise in the concentration of an anti-inflammatory cytokine demonstrate the effectiveness of MSC treatment to stop the cytokine storm brought on by SARS-CoV-2 infection.

Previous research has demonstrated that MSCs can force T-reg cells to differentiate, restrict T-cell proliferation, and induce T-cell death [146,147,148]. T-cells are also indirectly inhibited by MSCs’ effects on dendritic cells and NK cells [149]. Additionally, MSCs cause M1 macrophages to transform into anti-inflammatory M2 macrophages, which promotes tissue remodeling and inhibits the development of scar tissue [149,150,151]. These in vitro effects of MSC on immune cells could be either beneficial or detrimental, since at this moment, clinicians have described lymphopenia as one of the deleterious effects of SARS-CoV-2. What happens with our patients’ lymphocytes? Although limited by the small sample we were happy to see that CD4+ and CD8+ T lymphocytes number were recovered and, by an yet unknown mechanism, direct infusion of MSC solved partially the lymphopenia demonstrating that despite the big preclinical efforts in characterizing MSC responses, both in-vitro and inside the body, we are still learning on MSC profile and interaction with the body cells, tissues and fluids [95]. Such behavior demonstrates the adaptability of MSCs’ immunomodulation [149,152]. In this situation, MSC treatment may also be used to address COVID-19 infection’s long-term consequences, particularly those brought on by chronic inflammation. Even more, it gave us an additional reason to use them in combination with memory T-cells recovered from convalescent donors [13,15,71,153].

As potential novel medicinal therapies, MSCs have attracted a lot of attention. Clinical MSC therapy development is based on in-depth research using animal models of human illnesses and disorders that show improved results [154,155,156,157,158].

It is currently known that MSCs have a variety of physiological impacts, including preserving tissue homeostasis and promoting regeneration [159,160], in addition to the immunomodulatory effects appropriate for medical use [161]. Therefore, the scope of applications has been widened to embrace acute respiratory distress syndrome (ARDS), multiple sclerosis (MS), Crohn’s disease (CD), amyotrophic lateral sclerosis (ALS), and graft-versus-host disease (GVHD) [162,163,164,165].

Recent research published by Couto et al. [10] found 195 registered studies including 204 different cell-based therapeutics in numerous countries. The US led most of the trials with 53 trials, followed by China (43) and Iran (19). However, when adjusted for population, Israel, Spain, Iran, Australia, and Sweden had the most trials per million inhabitants. These studies focused mostly on multipotent MSCs, accounting for 72% of the trials. Other cell types included natural killer (NK) cells (9%), as well as mononuclear cells (6%). Of the 26 studies that reported data by July 2022, 24 used MSC infusions. A meta-analysis of these studies found that MSC treatment was linked to a lower risk of mortality from COVID-19, with a relative risk reduction of 0.63 (95% confidence interval: 0.46 to 0.85). This data suggests a possible advantage of MSC therapy, but the study also found substantial diversity in MSC suppliers, production procedures, and delivery protocols, emphasizing the need for standardized guidelines. A retrospective study on the long-term benefits of Cell Therapy on COVID-19 patients is needed.

## 8. Conclusions

After the pioneer studies from our group using adipose tissue-derived MSCs to treat critically ill COVID-19 patients intubated and mechanically ventilated, dozens of clinical trials were initiated (72%) that, in summary, report clear benefits. Whilst MSCs, from different sources, are mostly used, other cell types such as NK cells (9%), and mononuclear cells (6%). Furthermore, CD45RA−/CD45R0+ cells, introduced by our group, cannot be considered only antiviral since they trigger other immune cell responses [71]. Given the successful vaccination programs, the incidence and prevalence of COVID-19 diminished substantially, and a group of trials had to be suspended because of the lack of statistical potency reached.

A pooled analysis of the MSC studies revealed a noteworthy finding: MSCs exhibited a relative risk reduction for all-cause COVID-19 mortality, with a relative risk (RR) of 0.63 (95% CI 0.46 to 0.85). This outcome aligns with previous smaller meta-analyses, supporting the notion that MSC therapy may confer a clinical benefit for patients with COVID-19. Their effects on Long-COVID remain to be studied.

As compared with previous reported data, these are the better effects of an innovative treatment on mortality, complications, and short-term post-COVID-19 sequelae. Ours and other unpublished data demonstrate that a high proportion of COVID-19 survivors develop lung fibrosis. It cannot be excluded that anti-fibrotic MSCs may be used to recover full lung function in COVID-19 survivors.

Based on the observations and results of existing research, the use of cell therapy, particularly MSCs or MSC secretome, together with CD45RA-memory T-cells to treat COVID-19 looks promising. MSC treatment has demonstrated potential for cytokine storm suppression, immune system restraint, and lung damage healing during SARS-CoV-2 infection. The positive benefits supported by MSCs are notable, despite the fact that the mechanisms of action of these cells are not yet fully understood. Cell-based therapy might be viewed as an alternate treatment for managing the public health crisis, which includes epidemics in hospitals and care facilities and the breakdown of the medical infrastructure.

## Figures and Tables

**Table 1:** Etiopathology, symptomatology, and proposed treatments for long COVID.

Category	Etiopathology/Symptoms	Current Treatments	References
Immune Dysregulation	Autoantibodies, T-cell exhaustion, cytokine storms (e.g., IL-6, IFN-γ)	Immunomodulators (e.g., low-dose steroids, IVIG, biologics under trial) CD45RA- memory T-Cells RELEASE Trial-	EudraCT: 2020-001266-11, NCT04348461 [11,12,13,14,15,37,38,39]
Persistent Viral Reservoirs	COVID-19 remnants in tissues	Antivirals (e.g., Paxlovid in trials for Long COVID), immune-based therapies (suggested CD45RA-)	NCT05668091, [40]
Neurological Symptoms (Brain Fog, Fatigue)	Neuroinflammation, mitochondrial dysfunction	Cognitive therapy, graded exercise, mitochondrial support (e.g., CoQ10, B vitamins) Suggested MSC-Capilla Frontiers-)	[41]
Gut Microbiome Imbalance	Dysbiosis, leaky gut, inflammation, bacterial translocation	Probiotics, Nutraceuticals, dietary modifications, fecal microbiota transplants (in research)	[42]
Hormonal Dysregulation	HPA axis dysfunction, thyroid abnormalities	Hormone replacement (if indicated), lifestyle interventions	[43]
Respiratory Dysfunction	Lung inflammation, fibrosis, reduced oxygen uptake	Pulmonary rehabilitation, inhaled steroids, supplemental oxygen (if needed) MSC/ Secretome	[44]
Metabolic Dysfunction	Insulin resistance, hormonal imbalances (e.g., low cortisol)	Metformin (NCT04510194), hormone replacement therapy, lifestyle/diet change	[36]
Mitochondrial Dysfunction	Impaired energy production, oxidative stress	Antioxidants (e.g., CoQ10, NAC), metabolic support therapies/Nutraceuticals	[45]
Cardiovascular Issues	Myocarditis, arrhythmias, post-COVID heart failure	Cardiovascular monitoring, beta-blockers, ACE inhibitors	[46,47]

**Table 2:** Mechanism of Action (MOA). This schematic breakdown provides a detailed overview of the various mechanisms employed by MSCs, emphasizing their diverse and targeted actions in promoting tissue repair and regeneration.

**MSC Mechanism of action**	**Immunomodulation**	Inhibition of T-cell activation
		Suppression of T-cell proliferation
		Modulation of cytokine secretion
		Interaction with immune cells
	**Anti-Inflammatory**	Release of anti-inflammatory factors
		Inhibition of pro-inflammatory cytokines
	**Paracrine Signaling**	Secretion of trophic factors
		Release of growth factors
		Cytokine secretion
	**Exosome Release**	Bioactive molecules
		MicroRNAs
		Proteins
	**Angiogenesis**	Secretion of angiogenic factors
		Promotion of new blood vessel formation
	**Extracellular Matrix (ECM) Modulation**	Secretion of matrix metalloproteinases (MMPs)
		Tissue inhibitors of metalloproteinases (TIMPs)

## Data Availability

All data are included in the manuscript. Availability of data and materials is all included in the manuscript.

## Abbreviations

AMPK	adenosine monophosphate activated protein kinase
ARDS	Acute respiratory distress syndrome
ATMP	Advanced therapy medicine product
CBD	Cannabidiol
CBN	Cannabinol
CD	Cluster differentiation
CIBA	Consecutive Intra-Bronchial Administration
COPD	Chronic obstructive pulmonary disease
CRP	C-reactive protein
CT	Computerized tomography
DNA	Deoxyribonucleic acid
ECM	Extracellular matrix
ECMO	Extracorporeal membrane oxygenation
EUA	Emergency use authorization
FGF	Fibroblast growth factor
HGF	Human growth factor
HLA	Human Leucocyte Antigen
IDO	Indolamine-pyridole 2,3-dioxugenase
IFN	Interferon
IGF	Insulin-like growth factor
ITIMs	immunoreceptor tyrosine-based inhibitory motif
JAK	Janus kinases
LFA	Lymphocyte function-associated antigen
MCP	Monocyte chemoattractant protein
MHC	Major Histocompatibility Complex
MMP	Matrix Metalloproteases
MOA	Mechanism of Action
MSC	Mesenchymal stem/stromal cells
PACS	post-acute COVID symptoms
PCC	Post COVID Condition
PIFS	Post-Infective Fatigue Syndrome
ROS	Reactive oxigen substances
SDF	Stromal cell-derived factor
TGF	Transforming growth factor
THC	tetrahydrocannabinol
TIGIT	T-cell immunoreceptor with immunoglobulin and immunoreceptor tyrosine-based inhibitory motif domains
TIM	T-cell immunoglobulin domain
TIMP	Tissue inhibitors of metalloproteinases
Tmem	Memory T lymphocytes
TNF	Tumor necrosis factor
VCAM	Vascular cell adhesion protein
VEGF	Vascular endothelial growth factor
WHO	World health organization
